# Lifestyle Medicine in Medical Education: Benefits for Patients, Communities, and Our Future Medical Workforce

**DOI:** 10.1177/15598276251413215

**Published:** 2026-01-21

**Authors:** Stephen Russell Naulls, Anna Ogier, Ana Baptista, Christopher James Harvey, Richard J Pinder

**Affiliations:** 1 School of Public Health, Imperial College London, London, UK (SN, AO, AB, CH, RP); 2 Imperial College School of Medicine, Imperial College London, London, UK (SN, AO, AB, CH, RP); 3 Department of Clinical Neurosciences, Brighton & Sussex Medical School, Brighton, UK (SN); 4 Millview Hospital, Sussex Partnership NHS Foundation Trust, Hove, UK (SN)

**Keywords:** epidemiology, outcomes, training

## Abstract

Lifestyle Medicine (LM) is an increasingly important aspect of modern medical practice. LM focuses on sleep, healthy eating, physical activity, mental wellbeing, and the socioeconomic determinants of health. In 2019, Imperial College London introduced the ‘Lifestyle Medicine and Prevention’ (LMAP) module to educate medical students on these areas within a broader framework of public health ethics and practice. We hypothesised that early LM education may enhance students’ insight into their own health and wellbeing. Of 364 first-year medical students invited to complete an end-of-module survey, 278 consented to share their data and 239 responded to the open-ended question analysed: ‘Has your LMAP learning encouraged you to change any of your health behaviours? Please explain how’. Responses were analysed thematically. Of 239 responses, 155 students (66%) reported changes in their health behaviours after the LMAP module, 57 (24%) reported no change, and 27 (10%) described early contemplation of change. Lifestyle Medicine education may encourage reflection and positive health behaviour change amongst medical students.

Students described how the Lifestyle Medicine learning encouraged them to think about their own behaviours and that of their family, or a possible patient.

## Introduction

The field of Lifestyle Medicine has gained recognition as an integrative clinical discipline which utilises evidence-based lifestyle modifications to prevent, manage, and even reverse long-term conditions.^1^ While Lifestyle Medicine is increasingly recognised in medical practice across the world, its integration into medical education has been challenged by some, with debates surrounding its place and relevance.^2,3^

Traditional medical education in the west has prioritised anatomy, physiology, pharmacology, and clinical skills over wider domains such as preventive medicine and public policy.^4^ Introducing Lifestyle Medicine as a core discipline to undergraduate medical students presents an unusual challenge as it prioritises conceptual understanding across a wide array of population health sciences and critical-thinking over more conventional rote learning and competency-assessed clinical skills. However, recent research has suggested medical students find such content engaging and acceptable.^5,6^ Lifestyle Medicine recognises that the majority of the patient’s life exists outside the clinic. Lifestyle Medicine potentially represents an important vehicle for the paradigm shift needed towards person-centred care, shared decision-making, and holistic patient management.^1^

Lifestyle Medicine is conceptualised differently across the world.^1,7,8^ There is, nevertheless, consensus on its core domains of sleep, healthy eating, physical activity, and mental wellbeing. In the United Kingdom, where our study is based, the socioeconomic determinants of health, and their collective impact on longer-term health outcomes, are also included, as well as avoiding harmful substances.^1^ This runs parallel to recognising the broader contextual factors such as social, economic, environmental, and cultural influences on patient outcomes and behaviour, and employing effective communication strategies to facilitate behaviour change.^1,9^ While these concepts may seem intuitive at first glance, for inexperienced early-years medical students learning about risk modification and prevention may prompt re-examination of their contextual understanding of health, disease, and society.

Since 2019, all first and second-year medical students at Imperial College School of Medicine (ICSM) in London have been required to undertake an innovative course called Lifestyle Medicine and Prevention (LMAP).^10^ The two LMAP modules, which comprise the course, constitute 15% of students’ learning and assessment over the first two years of the 6-year undergraduate medical course.^10^ The learning approach employs a flipped-classroom model, utilising small group, near-peer-clinician-led tutorials that explore how broader health determinants influence health behaviours, health outcomes, and how these can be addressed in clinical practice.^10,11^ The course is founded on evidence-based public health ethics and practices. There are three overarching aims across the LMAP modules.

1. Build knowledge, understanding, and skills that improve patients’ health through the practice of Lifestyle Medicine.
2. Foster a population health mindset to recognise the broader context in which our communities live and NHS professionals work.
3. Develop insights into students’ own health and wellbeing that enable students to thrive in their medical training and career.

To achieve the three aims, LMAP encourages frequent reflection, prompting students to examine their own health behaviours as well as those of their patients, whether positive or negative. For instance, our group has previously described the impact that studying during the COVID-19 pandemic has had on the importance students place on Lifestyle Medicine.^5^

We hypothesised that a key benefit of early exposure to the content taught in LMAP is the opportunity to improve students’ own health behaviours – an issue that has received renewed focus considering consistent workforce pressures across the United Kingdom’s National Health Services.^12^ We explore this here through the lens of social constructivism with reference to two associated behaviour change theories. Social constructivism suggests that learning is shaped through social interactions and shared experiences, which aligns with behaviour change theories that emphasise the role of social and environmental influences on individual health behaviours. A leading model for understanding behaviour change is the Capability, Opportunity, Motivation Model of Human Behaviour Change (COM-B). The COM-B model is a framework for understanding behaviour change, which posits that behaviour (B) is the result of interactions between three components: Capability (C), Opportunity (O), and Motivation (M). Capability refers to an individual’s physical and psychological ability to perform a behaviour. Opportunity encompasses external factors that make the behaviour possible or prompt it. Motivation involves both reflective and automatic mechanisms that activate or inhibit the behaviour.^13^

According to the Transtheoretical (Stages of Change) Model, some individuals may exhibit ‘contemplation’ before undergoing behaviour change.^14^ In this context, contemplation refers to the stage in which an individual recognises the need for change and starts to consider the benefits and drawbacks of modifying their behaviour.^14^ At this stage, the person is aware of the problem and is thinking about making a change, but has not yet committed to acting. Contemplation involves weighing options, reflecting on past attempts at change, and beginning to form intentions to adopt new behaviours.^14^ Pre-contemplation is the stage in behaviour change where an individual is not yet considering making a change, often because they are unaware of the need for it or are in denial about the problem. People in this stage do not intend to take action in the foreseeable future and may be resistant to acknowledging the need for change.^14^ Pre-contemplation and contemplation take place before the subsequent stages of behaviour change which run sequentially from preparation to action, maintenance, and finally termination.^14^

Both the COM-B and Transtheoretical Model have been used to explore how and why individuals alter their health behaviours across the pillars of Lifestyle Medicine.^15-17^ This qualitative study evaluates how students make sense of Lifestyle Medicine learning in relation to their own health behaviours – the third aim of the LMAP modules – drawing on social constructivism to understand how this process is shaped by their social and educational environment. Social constructivism complements the COM-B and Transtheoretical Model by highlighting the role of social interactions and learning opportunities in shaping capability, opportunity, and motivation for behaviour change. The results are interpreted using the COM-B and Transtheoretical Models, recognising behaviours change as both a personal and socially influenced process.

## Methods

### Data Collection

A bespoke survey was designed for the study. It comprised predominantly open-ended questions to enable students to reflect on their educational experience within the module, alongside Likert-scale rating questions and demographic data collection.

### Procedure

At the conclusion of their first academic year in June 2020, students were invited to complete a brief questionnaire aimed at facilitating reflection on their attitudes toward and experiences with the LMAP module. Students could opt-in to have their responses to the open-ended questions included in research, with all responses remaining anonymous. Amongst the questions posed, students were asked how the module content had influenced their own health behaviours, if at all. The exact wording of the question was ‘Has your LMAP learning encouraged you to change any of your health behaviours? Please explain how it has or has not impacted your health behaviours’. This paper focuses on analysing the responses to this specific question.

### Data Analysis

A thematic analysis of the open-text was conducted following Braun and Clarke’s^18^ approach. The analysis was done inductively by three coders. Coder 1 (SRN) familiarised themselves with the data to code meaningful elements, identifying overarching themes at the semantic level. These themes were then reviewed and refined by coder 2 (AO). Finally, coder 3 (CJH) independently coded a sample of responses to validate the themes, acting as an auditor. NVivo software was used for the analysis (QSR International Pty Ltd. (2020)).

### Participants

Out of a possible 364 students, 278 consented to share their data for research purposes, and 239 provided responses to the question about their experience of the impact of LMAP on their own health behaviours.

### Ethics

The study received ethical approval from Imperial College London’s Faculty of Medicine Medical Education Ethics Committee (MEEC1920-181).

## Results

Of 239 responses to the question, 155 students (66.0%) stated that they had changed their health behaviours in response to the LMAP module. 57 students (24.2%) stated they had not changed any health behaviours. 27 students (9.8%) demonstrated signs of contemplation in their responses (Table 1). ‘Contemplation’ was coded in instances where individuals described increased awareness of the importance of a given health behaviour without explicitly describing a behaviour change, in keeping with the transtheoretical model. The demographics of the respondents are reported in Table 2.

**Table 1.** Breakdown of the Number and Percentage of Participants Describing Behaviour Change, No Behaviour Change, or Signs of Contemplation.

Description of Health Behaviours	n	%
Identification of behaviour change	155	66.0
Absence of identification of behaviour change	57	24.2
Signs of contemplation	27	9.8
Total	239

**Table 2.** Demographic Characteristics of Respondents to LMAP Survey (n = 239).

Age in years	Mean (Standard Deviation)	19.05 (0.78)
		%
Gender	Female	52.4
	Male	35.2
	Other	0.4
	Prefer not to say	0.7
	No response	11.4
Ethnicity	Asian	30.8
	White	21.2
	Mixed	18.7
	Other	11
	Black	2.2
	Prefer not to say	1.8
	No response	14.3
Identifies as coming from a background where University isn’t the norm	Yes	17.6
	No	66.3
	Prefer not to say	12.1
Holds a Previous Degree	Yes	2.6
	No	86.1
	No response	11.4

Of those students reporting change in their behaviour during the module, behaviour change in relation to sleep was the most frequently described (n = 82; 36.9%). Students were least likely to describe behaviour change in relation to finances (n = 11; 4.9%). 56 students reported behaviour change across more than one area of lifestyle medicine (Table 3).

**Table 3.** Breakdown Demonstrating, of the Total Number of References to Behaviour Change (n = 220), the Percentage of Which Were Described in Relation to Each Area of Lifestyle Medicine.

Area of Lifestyle Medicine	n	%
Physical activity	47	21.4
Financial wellbeing	11	5.0
Mental wellbeing	18	8.2
Healthy eating	50	22.7
Adequate high-quality sleep	82	37.3
Avoiding harmful substances	12	5.5
Multiple areas	56	25.5
Total	220	

In Table 4, we present a summary of the thematic analysis. Three groups were identified amongst students’ self-assessment of their own behaviour change: identification of behaviour change, absence of behaviour change, and contemplation.

**Table 4.** Themes and Sub-themes Identified From Open Text Response to Question ‘Has Your LMAP Learning Encouraged You to Change Any of Your Health Behaviours? Please Explain How It Has or Has Not Impacted Your Health Behaviours’.

Themes		Sub-theme(s)
Identification of behaviour change	1 - Altered Capability, Opportunity, or Motivation	1A - LMAP provided tools to change behaviour
		1B - Teaching provided motivation for behaviours change
	2 - Learning led to reflection	2A - Increased awareness of importance of given behaviour due to teaching
		2B - Relation to professional identity by considering patients, peers, and family members
		2C - Direct reflection on own health behaviours
	3 - Found teaching interesting and enjoyable	
Absence of behaviour change	4 - No requirement for behaviour change	4A - Already possessed the relevant knowledge
		4B - No underlying negative health behaviours to change
		4C - Reinforcement of good health behaviours that already existed
	5 - Ongoing barriers to behaviour change persisted	5A - Behaviour change is difficult to implement
		5B - External factors influencing behaviour continued to persist
		5C - Unsure how to change the behaviour in question
		5D - Lack of motivation for behaviour change
	6 - Not enough time for behaviour change to occur	
Contemplation	7 - Student exhibited signs of contemplation but did not describe behaviour change	

### Students Describing Behaviour Change

Three main themes were identified amongst students describing behaviour change in response to the LMAP module. The first theme was change in relation to Capability, Opportunity, or Motivation, as described by the COM-B model (theme 1, Table 4).

It probably has impacted my behaviour. Because we've learned about how behaviour change is facilitated, on a personal level I can think twice about why I'm doing certain things or getting influenced one way. The COM-B model of behaviour change is very interesting to think about. (SUR-261)

This theme breaks down into two sub-themes: the LMAP module providing students with the tools to change their own behaviours (theme 1A, Table 4), and teaching content and activities providing additional motivation for students to undergo behaviour change (theme 1B, Table 4):

When getting to university I was already trying to change these bad habits I had, and LMAP confirmed my issues and gave me tools to solve them. (Theme 1A, P-047)

From my money & wellbeing-based [studies], I have found strategies to improve my financial situation, that I wouldn't haven't considered otherwise e.g. financial budgeting apps. (Theme 1A, P-035)

… Learning about the dangers of leading a sedentary life definitely motivated me to pursue a more overall healthy lifestyle. Moreover, I have begun to cook a lot more, instead of buying pre-made food. Knowing the nutrients and the lack of preservatives in my food definitely feeds back into the overall improvement of my lifestyle. (Theme 1B, P-175)

The second theme in the responses from students describing behaviour change is that Lifestyle Medicine learning led to self-reflection (Theme 2):

Yes, I have changed my sleeping habits and begun to think more about sleep. Being more conscious of things is always the first step. I've also been trying to maintain about 30 minutes of physical activity per day (although this has been difficult recently). (Theme 2, P-083)

This theme can be further divided into three sub-themes: increased awareness about the importance of a given behaviour due to teaching (theme 2A, Table 4), reflection pertaining to the student’s professional identity in relation to patients, peers, or family members (theme 2B, Table 4), and direct reflection and application to the student’s own health behaviours (theme 2C, Table 4):

[After the nutrition learning] I am definitely more aware of the things I eat on a daily basis. I always thought about calories only – as long as I wasn’t eating too little or too many calories a day I would be fine, but I neglected the actual micronutrients in the foods I ate and realised I was eating lots of empty calories. (Theme 2A, P-130)

…it has made me consider the different aspects to good health, for example it has encouraged me to incorporate a variety of different forms of physical activity into my daily life, more than just targeted exercise in order to stay active. (Theme 2A, P-222)

It has made me consider my health choices a bit more since I've realised that if I can't do what I've asked my patient to do, then why am I thinking that they can do it too. (Theme 2B, P-028)

I'm feeling really inspired. I think my investment has also encouraged my parents, whose job is mostly sedentary, to be more active. They have now begun their own fitness challenge too! (Theme 2B, P-100)

Every session has made me reconsider my health behaviours because there tend to be activities that are personalised to us. (Theme 2C, PARTICIPANT-034)

It has made me more aware of my caffeine intake and how this can be affecting my sleep, and consequently I have made an active effort to drink less coffee. It has also made me more appreciative of my situation, both financially, socially and in regard to the fact that I am not from a minority background/experience racial discrimination. (Theme 2C, P-144)

The final theme identified from student responses was that the way the teaching was delivered, through a social constructivist approach, made the student more open to attempting behaviour change (theme 3, Table 4):

After discussions [in tutorials] about a lot of these topics, I analysed my own life …I also found that talking about mental health issues [with peers in tutorials] in the last term has made me feel more open to talking about when I'm struggling, as I found first year quite draining and didn't want to tell anyone. (Theme 3, P-166)

### Students Describing Contemplation

Some student responses provided evidence of contemplation in the absence of any description of clear behaviour change. Many of these responses indicate an increased ‘awareness’ of the importance of a particular behaviour without explicit description of a change in behaviour:

Well now I know that I should be doing 150 mins of moderate exercise per week...it hasn't changed that, but I am now aware of what I do. (Theme 7, P-112)

I think it has made me think a bit more about my sleep schedule. Although it has been difficult to change, it is something I am more aware of. (Theme 7, P-146)

It has made me think more about the effect of lifestyle factors on my own health and although I haven't made any drastic changes, I definitely think about my own health behaviours more. (Theme 7, P-060)

### Students Describing No Behaviour Change

Although the majority of students reported some aspect of behaviour change, a minority of students did not. The first theme identified was students reporting that there was no requirement for behaviour change. This theme can be broken down into three sub-themes: the student already possessed the knowledge in relation to a particular health behaviour (theme 4A, Table 4); the student expressed that there was no underlying health behaviour issues for them to address (theme 4B, Table 4); and the teaching simply reinforced pre-existing health behaviours the student was already pursuing (theme 4C, Table 4).

LMAP has not encouraged me to change any of my behaviours because most of the content I was already aware of and had included in my behaviours. (Theme 4A, P-195)

No as I wasn't struggling with any of the issues. (Theme 4B, P-017)

I don't think that it has encouraged me to change many of my behaviours, however it has reinforced my current 'good' behaviours, such as the importance of budgeting and being financially secure. (Theme 4C, P-020)

The second theme was the identification of ongoing barriers that persisted, preventing behaviour change from occurring (theme 5, Table 4):

It hasn't really changed my health behaviours. There are too many factors outside of public health statistics that influence my behaviours. (Theme 5, P-076)

This theme can be further divided into four sub-themes: behaviour change is difficult to implement (theme 5A, Table 4); external factors prevented the student from changing their behaviours (theme 5B, Table 4); ongoing uncertainty over how to change a given behaviour (theme 5C, Table 4); and lack of motivation for behaviour change (theme 5D, Table 4):

It has not really impacted my behaviours. Behavioural change, as discussed in LMAP is very difficult to implement and so predictably, I have not been able to implement any changes. (Theme 5A, P-011)

I don't feel that LMAP has had such a significant impact on me for me to actively pursue changing my habits […] other things take priority. (Theme 5B, P-093)

I realise the importance of changing some health behaviours I have – but dont really know how to. (Theme 5C, P-158)

I am unlikely to make changes to my life on the basis of my learning. Old habits die hard, unfortunately... (Theme 5D, P-073)

The final theme identified amongst students reporting no behaviour change was that behaviour change takes time to implement (theme 6, Table 4):

Although it has opened my eyes to a lot of poor habits of mine, I cant say it has resulted in a change (yet) as I believe it takes time and an active effort to really modify ones routine effectively. (Theme 6, P-133)

…it has made me realise that I need to have better sleep quality. for this change to actually start happening may take some more time. (Theme 6, P-269)

## Discussion

The findings from this study suggest that Lifestyle Medicine education and training in medical school is an important and potentially effective means of promoting positive health behaviours amongst medical students. Learning from near-peer educators is a powerful means of role modelling and influencing behaviour change.^19^ The minority of students who were unable to identify changes in their behaviour (or did not attempt to do so) may still benefit from increased empathy with patients to whom they will deliver Lifestyle Medicine advice in their future practice.

Our findings strongly suggest that the delivery of engaging and relatable Lifestyle Medicine teaching can foster health behaviour change amongst medical students. A substantial majority of medical students enrolled to the study described change in at least one health behaviour in response to LMAP teaching, and a large proportion described change across multiple areas. To our knowledge, this is the first study of its kind to demonstrate that Lifestyle Medicine teaching can result in health behaviour changes amongst a cohort of UK-based medical students. Contemporaneous research focussed on Lifestyle Medicine influencing healthcare students’ behaviours is sparse. In the United States, a pilot-study of a student-designed Lifestyle Medicine course, utilising aspects of experiential learning to leverage behaviour change, was implemented but took place outside of the formal curriculum and was not formally evaluated.^20^ At postgraduate level, a Lifestyle Medicine course in Israel, targeted towards a sample of 39 family medicine residents (the equivalent of a General Practice Specialty Trainee in the UK), was implemented.^21^ The authors reported a significant proportion of overweight residents increased their physical activity levels (from 12% to 21%) following the Lifestyle Medicine course.^21^

In our study, we have demonstrated that 66% of our participants described some type of behaviour change across various areas of Lifestyle Medicine, not limited to physical activity alone. Allowing for the clear limitation in extending this conclusion more broadly on the basis of a small study with limited sample size, collating these findings could demonstrate that the earlier stages of medical training may be a richer opportunity to influence healthcare professionals’ own health behaviours and build positive habits, coping strategies, and resilience, incorporating this into a student’s identity development as they progress through their career in medicine. Other studies have indicated that positive health behaviours may begin to decline towards the latter stages of postgraduate medical training, reiterating the possible importance of delivering such educational interventions early on in medical training to encourage positive habit formation and to normalise self-care, as is the case with the LMAP modules.^22,23^

The COM-B model can be employed to understand the changes in health behaviours described by students in this study. At its simplest, the psychological capability component of COM-B is essential to the changes to health behaviours described.^13^ Students, like the general population, cannot be expected to alter a behaviour if they have not received education that *X* behaviour is harmful, or *Y* behaviour is beneficial, for one’s health. This could explain why changes in health behaviours relating to sleep were most frequently reported by students. Generally, sleep medicine has received little coverage in medical curricula and has only recently been actively integrated into medical education, with coverage varying significantly between medical schools.^24^ Additionally, this is possibly the first time that medical students will have been targeted with public health messaging in relation to sleep.^25^ In contrast, other important areas of Lifestyle Medicine, namely physical activity and healthy eating, are covered comprehensively at earlier stages of formal education in the UK and internationally, and communicated more broadly to the general population through co-ordinated public health campaigns.^26,27^ Putting this together, it is possible that the focus on sleep medicine in the curriculum galvanised students to improve behaviours in this area because of greater psychological capability through education.

However, education alone is not always sufficient to alter human behaviour.^28,29^ Social constructivism is placed at the heart of LMAP tutorials and likely contributed to an environment conducive to behaviour change in keeping with the ‘Opportunity’ component of the COM-B model.^29^ The LMAP modules create a psychological safe space during reflective group-tasks in tutorials. In our constructivist learning environment, students are given the physical opportunity to access wider experiences and opinions through social interaction, which could be supplemented in moments of difficulty or disagreement by a near-peer (scaffolding) to enhance understanding, rather than passively receiving information didactically from an instructor.^30^ When discussing such challenges openly, students can see that others are facing similar barriers, which may normalise their experiences and reduce feelings of isolation often encountered when attempting behaviour change. The social opportunity for behaviour change is cultivated through the use of ‘LMAP Challenges’, endorsed by the Faculty, to encourage students to set a goal to improve their own behaviours in relation to a specific area of Lifestyle Medicine. Student responses expressed satisfaction with the role modelling provided by near-peers (resident doctors) involved in teaching the LMAP modules, through modalities such as ‘LMAP Challenges’. By having the whole year take part in this and it being endorsed by Faculty members, a greater onus is placed upon students to change their own behaviours – this dove-tailed with the physical opportunity provided for students with protected time in some tutorials to discuss progress against their goals in LMAP sessions, as described above.

The constructivist approach expands to the flipped-classroom nature of tutorials. The Zone of Proximal Development describes the gap between what a student can achieve independently and what they can achieve with guidance and support from a more knowledgeable other, such as a teacher, peer, or mentor.^30^ The flipped-classroom model empowers students to achieve as much as possible, independently, outside of the classroom environment (the active development area), then gives them access to peers and near-peers to refine their Lifestyle Medicine learning further, alongside any accompanying changes to health behaviours (the potential development area).^11,30^ In this respect, a social constructivist interpretation of these results suggests the LMAP module gave students access to greater physical and social opportunity to change their own health behaviours through access to peers and near-peers with whom they could discuss approaches to behaviour change across various pillars of Lifestyle Medicine, something they might otherwise not have access to.^30^

Finally, it is important to recognise the students who described no change in health behaviours, or for whom there was description of contemplation only. Students across all three groups (behaviour change, no behaviour change, and contemplation) described possible changes in the automatic and reflective component of the COM-B model, likely in response to the experiential learning components of the LMAP modules. Students described how the Lifestyle Medicine learning encouraged them to think about their own behaviours and that of their family, or a possible patient. This may represent a change in reflective motivation as students begin to change their values as they enter the slow transition from ‘student’ to ‘doctor’, enhanced by the experiential learning environment they are exposed to considering how to undergo behaviour change in their own lives.^31,32^ It is possible over time that the emotional responses and impulses a student experiences may change considering this new identity, leading to deeper changes in automatic motivation and habit formation in subsequent years.^13^ Some of the students themselves acknowledged that this could possibly be a lengthy process, as behaviour change is challenging and lengthy (theme 3, Table 4), and it is likely that only a longitudinal or prospective study would adequately capture this change over time. Nonetheless, students undergoing this level of mature and insightful reflection on their own health behaviours hints towards the role experiential learning should play in Lifestyle Medicine, as increasing students’ awareness of some of the challenges associated with behaviour change could be a powerful tool for establishing and maintaining empathy with patients they go on to provide Lifestyle Medicine advice to.^5^

This study presents notable limitations. The survey modality is subject to various biases including recall bias and recency bias. Measures regarding a perception of a change in health behaviour are all self-reported or inferred. The students were not followed-up longitudinally, so it is not possible to understand the extent to which this change is sustained over time, nor how this linked to subsequent measures of performance as a doctor and students’ wellbeing. The survey was also disseminated during the lockdown period of the COVID-19 pandemic – a time at which a possible confounder for changes in health behaviours is the heightened health-related anxiety present in society during that period.

To our knowledge, this is the first UK-based study to date to report how teaching medical students principles of Lifestyle Medicine affects students’ own behaviours. The LMAP modules at ICSM have demonstrated that teaching students about Lifestyle Medicine in engaging tutorials which embrace social constructivism, flipped-classroom tutorials, and experiential learning may encourage behaviour change amongst students in keeping with the COM-B model of human behaviour change. The extent to which this may benefit students in the long-term as they continue with medical training remains unclear.
